# A qualitative socio-ecological characterization of the plague threat at Hermelinda Market, La Libertad, Peru

**DOI:** 10.26633/RPSP.2017.107

**Published:** 2017-11-11

**Authors:** Ana Rivière-Cinnamond, Alain Santandreu, Guillermo Gonzalvez, Anita Luján, Marilú Noriega, John Omar Espinoza Quiroz, Yesenia Carpio, Jean-Marc Gabastou

**Affiliations:** 1 Health Emergencies Department Pan American Health Organization Washington, D.C. United States of America Health Emergencies Department, Pan American Health Organization, Washington, D.C., United States of America.; 2 Consorcio por la Salud Ambiente y Desarrollo Lima Peru Consorcio por la Salud, Ambiente y Desarrollo, Lima, Peru.; 3 Communicable Diseases and Health Analysis Department Pan American Health Organization Washington Communicable Diseases and Health Analysis Department. Pan American Health Organization, Washington, D.C.; 4 Gerencia Regional en Salud–La Libertad Gerencia Regional en Salud–La Libertad Trujillo Peru Gerencia Regional en Salud–La Libertad, Trujillo, Peru.

**Keywords:** Public health, plague, qualitative research, social determinants of health, Peru, South America, Salud pública, peste, investigación cualitativa, determinantes sociales de la salud, Perú, América del Sur, Saúde pública, peste, pesquisa qualitativa, determinantes sociais da saúde, Peru, América do Sul

## Abstract

**Objective:**

*To identify 1) the main determinants of persistent* Yersinia pestis *circulation and the associated threat of plague at Hermelinda Market—a large farmers’ market in the city of Trujillo, La Libertad, Peru—and the main actions taken against it, as perceived by local stakeholders; 2) the level of plague risk perception among local actors; and 3) recommended actions to solve the plague threat at the market*.

**Methods.:**

*A conceptual framework was developed combining a social determinants approach with a complex systems-thinking framework and a knowledge management perspective. A four-step qualitative protocol was carried out (literature review; stakeholder mapping; 37 semi-structured interviews; and coding/analysis). In the fourth step, the data collected in the semi-structured interviews were coded for eight social determinants of health (SDH) variables and analyzed with ATLAS.ti®, and an emerging category analysis was performed to identify risk perception levels*.

**Results.:**

*Based on analysis by SDH variable, the three main determinants of the plague threat at Hermelinda Market were: 1) local (Trujillo City) governance, 2) infrastructure and basic services, and 3) local culture. According to the same analysis, actions most frequently undertaken against plague involved 1) infrastructure and basic services, 2) social vigilance, and 3) communication. The emerging category analysis indicated local risk perception levels were low, with most of the data pointing to “unhygienic” (“naturalized”) lifestyles and a general lack of awareness about the disease prior to plague-related health concerns at the market as the cause*.

**Conclusions.:**

*The results indicate that the persistent circulation of* Yersinia pestis *at Hermelinda Market is not simply a technical matter but more of a managerial and cultural problem. As local governance was found to be a main factor in the persistence of this public health threat, future efforts against it should focus on sustainable inter-sectoral planning and education. Actions taken exclusively by the health sector and the improvement of infrastructure and basic services alone will not be enough to reduce the threat of plague at the market*.

## RESUMEN

**Objetivo.:**

Establecer: 1) los principales determinantes de la circulación persistente de Yersinia pestis y la amenaza asociada de peste en el mercado La Hermelinda, un gran mercado de agricultores en la ciudad de Trujillo, Departamento de La Libertad en Perú, y las medidas más adecuadas para combatir la bacteria, según la percepción de los interesados directos locales; 2) el grado de percepción del riesgo de peste entre los actores locales; y 3) las medidas recomendadas para resolver la amenaza de peste en el mercado.

**Métodos.:**

Se elaboró un marco conceptual que combinaba un enfoque de determinantes sociales con un complejo marco de pensamiento sistémico y una perspectiva de gestión del conocimiento. Se llevó a cabo un protocolo cualitativo de cuatro pasos (revisión bibliográfica; mapeo de interesados directos; 37 entrevistas semiestructuradas y codificación y análisis). En el cuarto paso, los datos recopilados en las entrevistas semiestructuradas fueron codificados conforme a ocho variables de los determinantes sociales de la salud y analizados con el programa ATLAS.ti ®, y luego se realizó un análisis de las categorías emergentes para establecer los grados de percepción del riesgo.

**Resultados.:**

Sobre la base del análisis de las variables de los determinantes sociales de la salud, los tres principales determinantes de la amenaza de peste en el mercado La Hermelinda fueron: 1) la gobernanza local (Ciudad de Trujillo), 2) la infraestructura y los servicios básicos y 3) la cultura local. Según el mismo análisis, las acciones emprendidas con mayor frecuencia contra la peste se vincularon con 1) la infraestructura y los servicios básicos, 2) la vigilancia social y 3) la comunicación. El análisis de las categorías emergentes indicó que los grados locales de percepción del riesgo fueron bajos y la mayoría de los datos apuntaron como causas a los modos de vida “antihigiénicos” (“naturalizados”) y a una falta general de concientización acerca de la enfermedad antes de la preocupación acerca de la salud relacionada con la peste en el mercado.

**Conclusiones.:**

Los resultados indican que la circulación persistente de Yersinia pestis en el mercado La Hermelinda no es sencillamente un asunto técnico sino sobre todo un problema cultural y de gestión. Como se encontró que la gobernanza local era un factor importante en la persistencia de esta amenaza a la salud pública, las actividades futuras para combatirla deben centrarse en la planificación y la educación intersectoriales sostenibles. Las medidas adoptadas exclusivamente por el sector de la salud y el mejoramiento de la infraestructura y los servicios básicos por sí solos no serán suficientes para reducir la amenaza de la peste en el mercado.

## RESUMO

**Objetivo.:**

Identificar 1) os principais determinantes da circulação persistente de Yersinia pestis e o risco associado de ocorrência da peste no Mercado Hermelinda, um grande mercado de produtos agrícolas no município de Trujillo, La Libertad, Peru, e as principais medidas adotadas para enfrentar esta situação, segundo a percepção dos interessados diretos locais, 2) o nível de percepção do risco de ocorrência da peste entre os atores locais e 3) as ações recomendadas para eliminar o risco de ocorrência da peste no mercado.

**Métodos.:**

Foi desenvolvida uma estrutura conceitual formada pela combinação de um enfoque de determinantes sociais, um enquadramento complexo de reflexão sistêmica e uma perspectiva de gestão do conhecimento. Foi conduzido um protocolo qualitativo de quatro etapas (revisão literária, mapeamento de interessados diretos, 37 entrevistas semiestruturadas e codificação/análise). Na quarta etapa, os dados coletados nas entrevistas semiestruturadas foram codificados para oito variáveis de determinantes sociais da saúde (DSS) e analisados com o programa ATLAS.ti®. Uma análise de categorias emergentes foi realizada para identificar os níveis de percepção de risco.

**Resultados.:**

A partir da análise segundo as variáveis de DSS, os três principais determinantes do risco de ocorrência da peste no Mercado Hermelinda foram: 1) governança local (município de Trujillo), 2) infraestrutura e serviços básicos e 3) cultura local. Segundo a mesma análise, as medidas adotadas com maior frequência para evitar a peste foram: 1) infraestrutura e serviços básicos, 2) vigilância social e 3) comunicação. A análise de categorias emergentes indicou que o nível de percepção local do risco de ocorrência era baixo, sendo que a maioria dos dados apontou como causa hábitos de vida anti-higiênicos (“naturalizados”) e falta geral de consciência da doença anterior às preocupações de saúde relacionadas com a peste no mercado.

**Conclusões.:**

Os resultados do estudo indicam que a circulação persistente de Yersinia pestis no Mercado Hermelinda não é simplesmente uma questão técnica, sendo mais um problema administrativo e cultural. Verificou-se que a governança local é um dos principais fatores para a persistência desta ameaça à saúde pública e os esforços futuros devem visar o planejamento intersetorial sustentável e a educação. Medidas adotadas exclusivamente pelo setor da saúde e a melhoria da infraestrutura e serviços básicos por si só não serão suficientes para reduzir o risco de ocorrência da peste no mercado.

Due to the resurgence of plague cases in recent years in Bolivia, Brazil, Ecuador, and Peru, plague has been given increased attention within the health systems framework of the Pan American Health Organization/World Health Organization (PAHO/WHO) (1).

Plague is caused by the telluric bacteria *Yersinia pestis* and has three clinical presentations: bubonic, septicemic, and pneumonic. Wild rodents are the reservoirs for the sylvatic cycle. Synanthropic rodents such as *Rattus rattus* and *R. norvegicus* are associated with the peridomestic cycle. The transmission mechanisms are bites from the rat flea *Xenopsylla cheopis*. Infected rodents develop the disease, and when they die of plague, carrier fleas search for other hosts. In that search, humans can get bitten and become infected with the disease.

A PAHO/WHO regional meeting of international plague experts was held in Lima on 22–24 January 2013 to identify a road map to address plague in South America. Meeting conclusions included the need to elaborate a strategic plan to control the disease—now known as the Strategic Plan for Surveillance and Control of Plague in Endemic Countries in South America. The Strategic Plan is grounded on three axes: 1) clinical and epidemiological aspects, 2) a laboratory component, and 3) social and environmental determinants. The objective was to reach zero human deaths and zero intra-domiciliary cases, as stated in PAHO Resolution CD49/R19 (2).

Most plague research has focused on epidemiologic, clinical, laboratory, and biologic aspects of the disease. Various studies have reported the improvements generated in each area over the last few decades (3–7). The social and environmental determinants of plague have been analyzed mainly from geographic, climatic, and agronomic perspectives, using geographic information system (GIS) techniques (1, 8–22). Although these studies are essential in identifying proxies for early warning and detection of plague cases, one crucial area in plague persistence has not been systematically addressed: its human-behavioral or social component, and its interaction with the ecologic sphere. Research exploring human behavior and risk perceptions and their link to ecological changes and the emergence of disease has been limited.

An increasing body of literature propounds the study of emerging and reemerging diseases from a broader perspective that considers behavioral, social, and ecological factors. Colwell first coined the term “biocomplexity” in 1999 in the context of cholera research (23). Other researchers have used different terms to describe similar concepts, including “social-ecological systems” (24, 25) and “human and natural systems” (26). In 2005, Wilcox & Colwell described the importance of the “interaction of humans and nature as a complex system” and proposed a “biocomplexity paradigm” as “a social-ecological approach for addressing and garnering and improving understanding of emerging infectious diseases” (27). In other, similar research, Wilcox & Gubler asserted that not only do ecological factors affect infectious disease emergence or reemergence but “the scale and magnitude of anthropogenic activity has reached a point of virtual co-dominance with natural processes of energy and material flows globally” (28). Using examples of reemerging diseases such as cholera or plague, both studies proposed research from a complex theory perspective on “how human behavior and ecosystems interact to contribute to disease emergence,” bridging “theory from the traditionally separate biological and social science disciplines” (27, 28). As asserted in the preface of a journal theme issue on climate change and vector-borne human diseases, “complex problems of human societies require new scientific approaches to better understand the fundamental drivers of their dynamics and enable interventions with appropriate policies” (29).

To address the need for a broader perspective in plague research, the third axis of the PAHO/WHO Strategic Plan included exploration of the effect of human behavior on plague persistence in endemic areas, and a methodology was developed to identify the social determinants of plague and risk perception at the local level. The selected site for the study for the socio-ecological characterization of the plague threat was a farmers’ market known as *Mercado La Hermelinda* (“Hermelinda Market”) in the city of Trujillo, in the department of La Libertad, Peru. This site was selected for the study because official epidemiologic surveillance from Peru’s National Institute of Health (*Instituto Nacional de Salud*, INS) had confirmed the circulation of *Yersinia pestis* among rodents at the market.^4^

## HERMELINDA MARKET (TRUJILLO, PERU)

**FIGURE 1.**
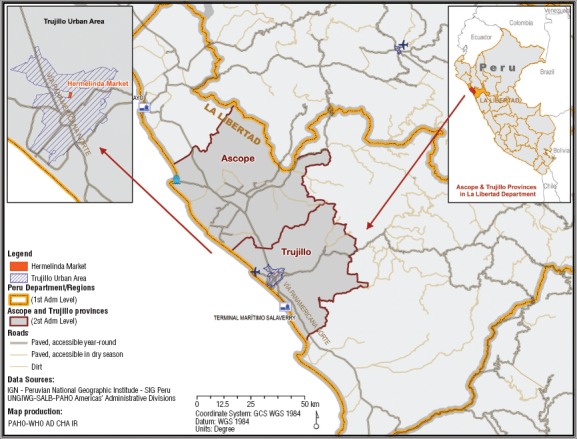
Map of Hermelinda farmers’ market in Trujillo City, Salaverry Harbor, and surrounding areas with intensive agriculture in Ascope and Trujillo Provinces, La Libertad, Peru, 2014

***Source:*** Prepared by PAHO-WHO based on data from: Peruvian National Geographic Institute (IGN); Special Interest Group (SIG) Peru; United Nations Geographic Information Working Group (UNGIWG); and SALB-PAHO (Second Administrative Level Boundaries project) Americas’ Administrative Divisions.

The urban area surrounding Hermelinda Market—Trujillo City—is the third-largest in Peru, with a population of 788 236 (30). It is located 34 meters above sea level on the coast of Trujillo Province, near the country’s second-largest international port, *Terminal Marítimo Salaverry* (“Salaverry Harbor”), and surrounded by rural, semi-arid agricultural areas with intensive sugar cane production (Figure 1). Trujillo Province has a total population of 811 979, of which 2.4% is rural and 3.0% is at the poverty level. Due to the Humboldt stream, temperatures at Hermelinda Market range from 14°C to 30°C. Rain is scarce (annual mean of 99 mm) and concentrated in the months of January to March. The characteristics of the area support Schneider et al.’s assertion that “plague occurs primarily in semi-arid” areas (1). The INS has confirmed *Yersinia pestis* circulation in synanthropic rodents at both Hermelinda Market and Salaverry Harbor, as well as Ascope district, in the adjacent province of the same name (Figure 1), a plague-endemic area—with human cases, including human fatality cases, as of 2013^4^—typical of Hermelinda Market’s surrounding environment.

Hermelinda Market opened in October 1987 and is one of the largest farmers’ markets in Peru’s North macro-region (a geographic area that includes the northern regions of Amazonas, Ancash, Cajamarca, La Libertad, Lambayeque, Piura, San Martín, and Tumbes). With more than 5 000 visitors per day, an area of 10 hectares, and 1 370 stalls, the market generates about US$ 300 000 per day (personal communication, M. Noriega, Regional Health Department, La Libertad). Producers from the North macro-region, including migrant workers from plague-productive areas in the Peruvian Andes, often convene at the market, which has limited water and sanitation facilities and a weakly articulated solid waste removal plan with the city of Trujillo. Based on the unpublished trap data for 2013 from the INS,^4^ plague reservoirs *Rattus rattus* and *R. norvegicus* are ubiquitous at the market, with *R. rattus* the most prevalent (Table 1). The flea vector *Xenopsylla cheopis* is also prevalent, with a specific index (SI)^5^ ranging from 0.8 to 7.71 for different sections of the market. Areas with an SI > 1 for *X. cheopis* are considered “at risk” for plague (31).

Given Hermelinda Market’s location in a highly populated urban area (near an international port, and intensive agriculture areas), and its large number of visitors and workers, GERESA–La Libertad, the regional health authority, declared a sanitary alert for the market and its surrounding area in January 2013 in order to implement rodent and flea control measures. Discussions were held to identify possible strategies for improving the market’s unsanitary conditions. The research presented below was conceived and designed to help guide those potential strategies. The specific aim of the study was to identify 1) the main determinants of persistent *Yersinia pestis* circulation and the associated threat of plague at Hermelinda Market, and the main actions taken against it, as perceived by local stakeholders; 2) the level of plague risk perception among local actors; and 3) recommended actions to solve the plague threat at the market.

**TABLE 1.** *Yersinia pestis* circulation among rats at Hermelinda farmers’ market based on testing of trap samples of the rat-flea vector *Xenopsylla cheopis*, Trujillo, La Libertad, Peru, 2013

Market section	Trap index^a^	Specific index^b^ for *X. cheopis* ^c^		Circulation of *Y. pestis* ^d^
		Rattus rattus	R. norvegicus	
Potatoes	6.7	7.71	5	Positive
Fruit	5	3.5	1	–^e^
Meat	23.3	1.53	–	Positive
Onions	10	3.72	–	Positive
Groceries	0	–	–	–
Merchandise	3.3	4.6	2	–
Live poultry	30.8	5.61	4	Positive
Wood/corn	11.7	2.85	0.8	Positive

***Source:*** Instituto Nacional de Salud. Study of *Y. pestis* circulation at La Hermelinda market (unpublished). Lima: INS; 2013.

a Number of traps with rats divided by total number of traps deployed, multiplied by 100 (31).

b Number of fleas of one specific species divided by total number of rodents of one specific species.

c A specific index (SI) > 1 for *X. cheopis* indicates risk of plague (31).

d Based on testing of *X. cheopis* trap samples.

e Data not available.

## MATERIALS AND METHODS

### Conceptual framework

The study’s conceptual framework and methodology as well as the tools used in its application were based on a combination of different approaches. These included 1) a social determinants of health (SDH) approach, 2) an ecohealth approach, and 3) a knowledge management approach.

#### SDH approach.

The SDH approach used in the analytical foundation for the research was based on the perspectives provided by 1) Whitehead & Dahlgren, who evaluated SDH in terms of the level of individualization or attribution (32, 33), and 2) Diderichsen et al., who considered SDH organized on the basis of “structural determinants” associated with policies and “intermediate determinants” directly affecting individuals (34). Analysis using these approaches allowed for the identification of social determinants of plague applicable to the Hermelinda Market, the study area. The identified social determinants, which served as variables for the qualitative analysis using ATLAS.ti®, included: 1) local (municipal) governance/political responsibility; 2) infrastructure and basic services (sanitation and hygiene); 3) local culture/popular knowledge; 4) social vigilance (social or community responsibility, social surveillance); 5) technical-scientific knowledge/research; 6) national governance; 7) communication and sensibilization; and 8) local economy and work. In addition, an emerging category analysis was performed to identify risk perception levels among the 37 people included in the semi-structured interviews described below.

#### Ecohealth approach.

The ecohealth approach was fundamental to the study because it combines complex and systems-thinking theory with transdisciplinary research and social participation. This combined approach is designed to produce results with higher social equity and improved social and environmental sustainability rates, and contributes to actual action research (35, 36).

#### Participatory knowledge management approach.

The participatory knowledge management approach (37, 38) focuses on the learning process in contexts of complexity and uncertainty. This approach allows for the construction of collaborative learning for change as part of a systematic process of compiling, processing, and critically analyzing individual and social information and socially relevant knowledge. Results can include not only tangible outcomes but also cognitive behavioral changes.

### Data collection and analysis

Between November 2012 (the onset of the regional health department’s sanitary alert at Hermelinda Market) and December 2014, a qualitative data collection and analysis protocol was carried out consisting of four main steps: 1) literature review; 2) stakeholder mapping; 3) semi-structured interviews; and 4) data coding/analysis.

#### Literature review.

Traditional search methods were used to generate content for the literature review, including database searches for published data and requests to key informants for relevant unpublished data. A total of 71 documents were obtained, including five laws, two norms, one official guideline from the Ministry of Health, eight resolutions, 41 press releases, and 14 journal articles. The information was used to better understand the context of plague in the study area, the applicable legal framework, and the treatment by the media of the sanitary alert.

#### Stakeholder mapping.

Based on discussions with local actors (market vendors, municipal health and other government authorities, etc.), key stakeholders involved in the plague problem at Hermelinda Market were identified, analyzed, and mapped (stratified) by 1) sector (public, private, or civil society) and 2) level of participation in decision-making (“control,” “influence,” or “interest”). Information was also gathered about stakeholder attributes and relationships with GERESA–La Libertad (strength and direction of influence). Due to the transdisciplinary and participatory nature of the mapping research, an ecohealth approach was used. The final list of key stakeholders (a total of 44) provided the sample pool for the semi-structured interviews.

#### Semi-structured interviews.

The number of interviewees (37 out of a pool of 44) was defined using the snowball technique. Semi-structured interviews were conducted and recorded and the results transcribed into a Microsoft Word document. The methods for compiling, processing, and analyzing the data (by individual stakeholder, and overall) were based on the knowledge management approach. Institutional review board approval and participant consent were not required because no personal data or medical histories were collected.

#### Coding/analysis.

The content of the 37 transcribed interviews was coded for one or more of the eight determinants of plague described above (local governance; infrastructure and basic services; local culture; social vigilance; technical-scientific knowledge; national governance; communication; and local economy) using ATLAS.ti® software (Scientific Software Development GmbH, Berlin, Germany). The analysis was designed to identify 1) the most important determinants of the plague threat at Hermelinda Market; 2) the top three areas of action taken against the plague threat thus far; and 3) other areas of action that might require special attention (by characterizing/grouping potential secondary effects of weaknesses in the main determinants). An emerging category analysis was also carried out (using ATLAS.ti®) to identify interviewees’ plague risk perception levels, using Elliot’s method (differentiating “aprioristic concepts” from “awareness raising concepts” by posing comparisons between “objective” situations and “socially constructed” perceptions) (39).

## RESULTS

### Stakeholder mapping

**FIGURE 2.**
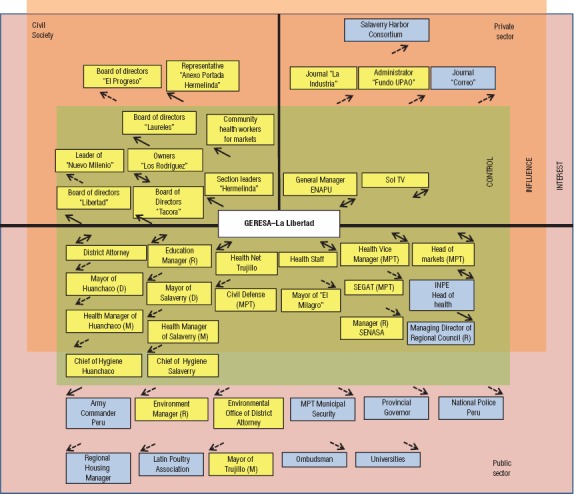
Mapping of key stakeholders involved in the plague problem at Hermelinda farmers’ market (*n* = 44) by sector (public, private, civil society); level of participation in decision-making (“control,” “influence,” “interest”); and strength and direction of relationships with GERESA,^a^ Trujillo, La Libertad, Peru, 2014^b^

***Source:*** Compiled by the authors based on the study results.

^a^*Gerencia Regional en Salud*, the health department of the Regional Government of Trujillo, and the main source of health-related decision-making for Hermelinda Market.

^b^ “Control over market decision-making” (green shading); “capacity to influence it” (orange shading); or “some interest in influencing it” (pink shading). Blue shading = secondary actors; yellow shading = key actors; light green shading = xxxxxxxx; D = department; R = regional; P = province. Relationships with GERESA: broken- versus solid-line arrows indicate strong versus weak relationships; one- versus two-sided arrows indicate top-down/bottom-up versus bidirectional relationships. As shown, GERESA had strong relationships with stakeholders in municipal Health (staff and managment) as well as Education, Legal, and TV, the national port authority at Salaverry Harbor (*Empresa Nacional de Puertos*, ENAPU) , the owners of one section of the market (“Corrales Los Rodríguez”), and the head (supervisor) of markets for Trujillo City (MPT). UPAO: *Universidad Privada Antenor Orrego*; MPT: *Municipalidad Provincial de Trujillo*; SEGAT: *Servicio de Gestión Ambiental de Trujillo*; SENASA: *Servicio Nacional de Sanidad Agropecuaria*; INPE: *Instituto National Penitenciario*.

The results of the stakeholder mapping are shown in Figure 2. A total of 27 key stakeholders were viewed as having control over decision-making to address the plague problem at Hermelinda Market (green shading); six were viewed as having the capacity to influence it (orange shading); and 11 were viewed as having some interest in influencing it (pink shading). The data collected also allowed for the characterization of stakeholder attributes (not shown) and relationships with GERESA–La Libertad (strong versus weak and the direction of influence, depicted respectively with solid- or broken-line and one- or two-sided arrows).

According to the mapping data, which were based on 37 local actors’ responses from the semi-structured interviews, 1) the public-sector stakeholders, specifically those related to the health sector, had the most influence on (“control” over) decision-making; 2) the strongest relationships with GERESA–La Libertad (in terms of level of influence and bidirectionality) were with the municipal Health sector (staff and management) as well as stakeholders in Education, Legal, and TV, the national port authority at Salaverry Harbor (*Empresa Nacional de Puertos*, ENAPU), the owners of one section of the market called “Corrales Los Rodríguez,” and the public sector head (supervisor) of markets for Trujillo City (*Municipalidad Provincial de Trujillo*, MPT); 3) the relationships between GERESA–La Libertad and actors in other sectors, including most local (city) government, were was not strong; and 4) the relationships between GERESA–La Libertad and most civil- and private-sector stakeholders, particularly including the market section/vendor boards and other representatives, were weak (one-directional), as shown by the arrows in Figure 2.

### Main determinants of the Hermelinda Market plague threat

Based on the analysis performed with ATLAS.ti®, the eight main determinants of the plague threat at Hermelinda Market are shown in Table 2 in order of importance. The three most important determinants were: 1) local (Trujillo City) governance, 2) infrastructure and basic services, and 3) local culture.

**TABLE 2.** Social determinants of plague at Hermelinda farmers’ market by importance as a cause based on the number of mentions by 37 key stakeholders in semi-structured interviews, Trujillo, La Libertad, Peru, 2014

Social determinants of plague (in order of importance)	No. of mentions by stakeholders
1. Local (municipal) governance/political responsibility	138
2. Infrastructure and basic services (sanitation and hygiene)	115
3. Local culture/popular knowledge	103
4. Social vigilance (social or community responsibility, social surveillance)	97
5. Technical-scientific knowledge/research	88
6. National governance	88
7. Communication and sensibilization	79
8. Local economy and work	38
Total	746

***Source:*** Compiled by the authors based on the study results.

**FIGURE 3.**
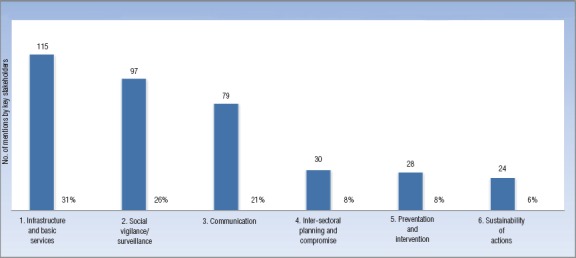
Areas of action to fight plague at Hermelinda farmers’ market based on number of mentions by 37 key stakeholders in semi-structured interviews, Trujillo, La Libertad, Peru, 2014

***Source:*** Compiled by the authors based on the study results.

### Recommended areas of action

The ATLAS.ti® analysis also showed that the areas of action most frequently addressed in efforts to control the plague threat at Hermelinda Market were 1) infrastructure and basic services, 2) social vigilance, and 3) communication. Characterizing and categorizing the potential effects of weakness in “local culture” supported the use of communication, to help address low risk perception. Three additional areas of action were identified by characterizing the potential effects of weakness in “local governance” and “infrastructure and basic services” (i.e., mismanagement): 1) prevention and intervention activities, 2) sustainability of actions, and 3) inter-sectoral planning and compromise. All six recommended areas of action against plague are shown in Figure 3 by level of importance, based on the ATLAS.ti® analysis of the stakeholder interview data.

### Low risk perception

Coding and classifying risk perception in the emerging category analysis (using ATLAS.ti®) showed low levels among the local actors that stemmed mainly from 1) unhygienic (“naturalized”) lifestyles (living with animals inside the household, high density of persons within household, low personal hygiene, storing unwrapped food in the household, etc.) and 2) a general lack of awareness (e.g., most knowledge about plague among market actors came only from their recent experience with plague-related events rather than any preexisting knowledge about the disease). Other variables that emerged as potential factors in the low risk perception found in the analysis were 1) level of education; 2) inclusion of plague in the political agenda (i.e., recognition of plague as a public health issue—plague is not considered a public health problem in La Libertad so is not included in the political agenda of the regional or local authorities); and 3) ability to identify all plague determinants (i.e., full awareness of all determinants affecting the occurrence of plague).

The data that were analyzed (collected in the stakeholder interviews) included explicit mentions of the presence of rodents and fleas in the market associated with (and coded for) the “unhygienic lifestyles” category (e.g., “We have seen rats in the market”; “Rats passed in front of us, and there were lots of fleas that were biting all of us”; and “People have rats around and do nothing”). The lowest risk perception levels were associated with both the “unhygienic lifestyles” and “behavior change/knowledge based only on recent exposure to a plague event” categories.

## DISCUSSION

According to Ellis & Wilcox, “potentially broadly effective upper level interactions and processes (e.g. inter-sectoral coordination) may be negated by lower level phenomena (e.g. insecticide resistance, behavioral changes, individual commitment)” (40). In the Hermelinda Market ecosystem, both upper- and lower-level phenomena seem to be inefficient in reducing the threat associated with the circulation of *Yersinia pestis*, generating a negative synergy, with weak inter-sectoral coordination further fostered by individuals’ reluctance to change behavior and lack of commitment. These study results suggest three main determinants of Hermelinda Market’s plague threat that must be addressed before progress can be made in solving it: local (municipal) governance; infrastructure and basic services, and local culture.

### Local governance

Deficits in local governance are a root cause of plague at the market due to three main factors: 1) the multi-sectoral nature of the plague threat, which is not solely associated with the health sector and thus requires actions in other sectors (e.g., housing and infrastructure; water, sanitation, and hygiene; education; employment; and agriculture) to prevent plague cases as well as endemicity; 2) weakness in the capacity for multi-sectoral implementation at the local level, due to budget planning that remains mostly vertical, which does not allow for a transdisciplinary approach; and 3) lack of sustainability in the implementation of concerted actions, due to a shortsighted planning process focused mainly on emergency situations and local aid politics (with financial assistance allocated only in emergencies). The importance of these issues with regard to local governance was corroborated by the results of the analysis showing that secondary areas of action requiring attention included “planning and inter-sectoral compromise” and “sustainability.” The analysis also showed that the multi-sectoral commission created to make decisions and take action on *Yersinia pestis* circulation in the market included delegates who were designated by their superiors but had no decision-making power. Therefore, even if the commission was meeting periodically, problem-solving actions were not decided upon in a dynamic and assertive manner, or implemented.

### Infrastructure and basic services

Problems associated with “infrastructure and basic services” are rooted in 1) weak planning and coordination between Trujillo Province and Trujillo City government, particularly in market solid waste management and disposal, and water and sanitation service provision, and 2) inadequate practice/acceptance of existing infrastructure and hygiene standards for stalls, including those on overcrowding and the coexistence of animals among shopkeepers.

### Local culture

Issues related to “local culture” were deeply entrenched with 1) low levels of plague risk perception among shopkeepers, who demonstrated “unhygienic lifestyles” despite intense health education campaigns focused on plague prevention carried out by GERESA–La Libertad; 2) a generally low education level; and 3) a lifestyle in which economic incentives outweighed sanitary standards in the context of a rural migrant workforce that lived with solid waste, food, and animals in overcrowded and substandard housing. Similar lifestyles have been identified in other countries/cultures with plague outbreaks (5).

Institutional factors combined with the increase in population, human density, mobility, and modes of transportation have led to a situation whereby “in the same period and places in which most significant human-environmental transformation have been taking place in recent history, divestment in public health infrastructure, including ineffective hygiene and diseases control measures, has also been occurring” (27). Therefore, the combination of the “local culture” determinant, the identified low level of risk perception of the market shopkeepers, and the inefficiency in coordinating the implementation of “infrastructure and basic services” suggests a scenario similar to the one presented by Horwitz & Wilcox, which highlights the inextricability of the host-vector-pathogen complex (41), whereby classically categorized vector prevention and control strategies are embedded in a “human-built” environment (42) with the potential to limit their empirically tested effectiveness and efficiency. With this perspective in mind, “even human–human interactions and behavior, although not traditionally the topic of ecological research, can be viewed as ecological in nature” (40).

“Urbanization, agricultural intensification, and habitat loss and alteration, in particular, driven by population growth and consumption, characterize the model” (27) in which social drivers lead to regional environmental change and public health policy failure if not adequately planned and coordinated. Hermelinda Market demonstrates most of those social drivers, depicting, in an urban setting, the effects of agricultural intensification around Trujillo, where intensive sugar cane production is projected to increase (from 630 000 hectares) as part of the Chavimochic irrigation project along the Peruvian northern coastline, which will demand 120 000 extra workforce (personal communication, M. Noriega, GERESA–La Libertad). This type of man-made manipulation of the natural environment has been identified as broadening plague-related rodents’ habitat, generating an increased risk for plague (19). Unhygienic transportation of products to Hermelinda Market through this type of landscape will likely increase the probabilities of higher rodent and flea indexes in the market. This, combined with the expected increase in the migrant workforce, its cultural habitudes and low risk perception levels, such as living with guinea pigs (*Cavia porcellus*) (43), and the overcrowded habitat of the market, adds another hazard to the plague critical control points risk identification chain. It seems clear that the three main determinants of the plague threat identified in the context of the study of Hermelinda Market should be addressed, given the 1) geographic location of the market (the city of Trujillo, the third-largest urban area in Peru); 2) its number of visitors per day (more than 5 000); and 3) its proximity to the international port, Salaverry Harbor.

### Limitations

This study could have been improved by 1) reproducing the methodology in a plague-endemic, rural, intensive agriculture setting where human cases had been identified in the recent past; 2) improving the risk perceptions identification technique to include quantitatively measureable results; 3) including a “social networks analysis” to identify the most effective channels to convey information for problem-solving; 4) gathering descriptive data to allow for statistical analysis; 5) performing a comparative molecular characterization, and virulence studies, of the circulating *Yersinia pestis* strains in Hermelinda Market, Salaverry Harbor, and the endemic rural setting of Ascope; and 6) including more ecological information in the model, to ultimately allow for a true complex systems-thinking geographic map of social, ecological, and epidemiological variables.

### Recommendations

To address the perceived weaknesses in “local governance,” the authors recommend that the city of Trujillo take the lead in the planning, coordination, and articulation of the actions specified in the Strategic Plan for Surveillance and Control of Plague in Endemic Countries in South America according to the Hermelinda context. This would facilitate local (municipal) authorities’ engagement with and appropriation of the Strategic Plan as well as the actions of the participating sectors (health, education, agriculture, housing and infrastructure, and employment). The planning process should be led by a commission with specially delegated decision-making members and a horizontal, cross-sectoral budgeting process.

The implementation of the Strategic Plan in Peru’s northern macro-region would ensure institutional continuity in actions scheduled and performed to address weaknesses in the “infrastructure and basic services” determinant, including improving access to water and sanitation and adequate solid waste management at the market, and better enforcement of norms and legislation related to physical and hygienic conditions, as well as standards, at the stall and market level.

Implementation of the Strategic Plan should be intertwined with a different approach to health education. This study showed that the local culture at Hermelinda Market is the basis of social behavior with regard to health, and even when intensive health education programs focused on plague prevention in the market were carried out by GERESA–La Libertad, risk perception levels remained low and did not translate into behavioral changes among local shopkeepers or market consumers. More cohesion among shopkeepers might be fostered though the creation of an associative mechanism to convey concerted messages to the local (municipal) authorities.

### Conclusions

This study found that in addition to technical issues related to infrastructure (e.g., water and sanitation), weaknesses in local (municipal) governance, local culture (specifically, low plague risk perception levels), and inter-sectoral coordination contribute to the plague threat at Hermelinda Market. Therefore, the problem is not only technical in nature but also has managerial aspects, so actions taken exclusively by the health sector, and/or improvement of the infrastructure and basic services, will not be enough to reduce this public health threat.

### Disclaimer.

Authors hold sole responsibility for the views expressed in the manuscript, which may not necessarily reflect the opinion or policy of the RPSP/PAJPH or the Pan American Health Organization (PAHO).

## Footnotes

4 Instituto Nacional de Salud. Study of *Y. pestis* circulation at La Hermelinda market (unpublished). Lima: INS; 2013.

5 Number of fleas of one specific species divided by total number of rodents of one specific species.
